# Ketoacidosis associated with tirzepatide use in a nonobese individual without diabetes

**DOI:** 10.1210/jcemcr/luag054

**Published:** 2026-04-08

**Authors:** Ryosuke Kemmotsu, Tomonori Okita, Naoki Koike, Ryoko Inui, Yukiko Tabuchi, Tetsuyuki Yasuda

**Affiliations:** Department of Diabetes, Endocrinology and Metabolism, Osaka International Medical and Science Center (Osaka Keisatsu Hospital), 2-6-40 Karasugatsuji Tennoji-ku, Osaka 543-0035, Japan; Department of Diabetes, Endocrinology and Metabolism, Osaka International Medical and Science Center (Osaka Keisatsu Hospital), 2-6-40 Karasugatsuji Tennoji-ku, Osaka 543-0035, Japan; Department of Diabetes, Endocrinology and Metabolism, Osaka International Medical and Science Center (Osaka Keisatsu Hospital), 2-6-40 Karasugatsuji Tennoji-ku, Osaka 543-0035, Japan; Department of Diabetes, Endocrinology and Metabolism, Osaka International Medical and Science Center (Osaka Keisatsu Hospital), 2-6-40 Karasugatsuji Tennoji-ku, Osaka 543-0035, Japan; Department of Diabetes, Endocrinology and Metabolism, Osaka International Medical and Science Center (Osaka Keisatsu Hospital), 2-6-40 Karasugatsuji Tennoji-ku, Osaka 543-0035, Japan; Department of Diabetes, Endocrinology and Metabolism, Osaka International Medical and Science Center (Osaka Keisatsu Hospital), 2-6-40 Karasugatsuji Tennoji-ku, Osaka 543-0035, Japan

**Keywords:** tirzepatide, euglycemic ketoacidosis, glucose-dependent insulinotropic polypeptide (GIP), lipolysis, off-label use

## Abstract

Euglycemic ketoacidosis is metabolic acidosis with elevated ketone bodies despite normal or mildly elevated glucose levels and has rarely been reported in nonobese individuals without diabetes using tirzepatide. A 23-year-old Japanese woman without obesity presented with a 4-day history of nausea, vomiting, and diarrhea after the second self-administered dose of tirzepatide (2.5 mg) obtained through an aesthetic clinic without ongoing medical supervision. She had no history of diabetes, alcohol consumption, or carbohydrate restriction. Laboratory results showed high anion gap metabolic acidosis (anion gap: 25.9 mmol/L) [normal reference range (ref): 8-16 mmol/L], elevated serum β-hydroxybutyrate (5.5 mmol/L) (ref: <0.07 mmol/L) and acetoacetate (1.4 mmol/L) (ref: <0.01-0.07 mmol/L), with concomitant metabolic alkalosis compatible with vomiting and mild hyperglycemia (196 mg/dL [SI:10.9 mmol/L]) (ref: 70-100 mg/dL [SI: 3.9-5.6 mmol/L]). No definitive infectious source was identified on evaluation, and euglycemic ketoacidosis was diagnosed. She received intravenous fluids containing glucose, electrolytes, and continuous insulin infusion, which rapidly corrected the acidosis. Her symptoms resolved within several days, and follow-up evaluation confirmed normal glucose tolerance. This case adds to the limited clinical evidence of tirzepatide-associated euglycemic ketoacidosis in a non-obese individual without diabetes. Caution is warranted regarding the off-label use of tirzepatide for aesthetic weight reduction.

## Introduction

Euglycemic ketoacidosis is characterized by a high anion gap metabolic acidosis accompanied by elevated serum ketone bodies in the absence of hyperglycemia, defined as plasma glucose levels of < 250 mg/dL (SI: 13.9 mmol/L) [1]. It arises when relative insulin deficiency and increased glucagon secretion enhance lipolysis and hepatic ketogenesis, despite normal or mildly elevated plasma glucose levels. Common scenarios include reduced carbohydrate intake, prolonged fasting, acute illness, surgery, or insulin dose reduction in individuals with diabetes treated with sodium-glucose cotransporter 2 (SGLT2) inhibitors, as well as starvation, pregnancy, or excessive alcohol consumption in individuals without diabetes [1, 2].

Tirzepatide, a dual agonist of glucagon-like peptide-1 (GLP-1) and glucose-dependent insulinotropic polypeptide (GIP) receptors, has recently been introduced worldwide for the treatment of type 2 diabetes mellitus and obesity; it has potent effects on glycemic management and weight reduction [3-6]. However, in Japan, tirzepatide has also been used off-label for aesthetic weight reduction among individuals without obesity or diabetes, despite the lack of safety data in this population. We describe a rare case of euglycemic ketoacidosis in a non-obese woman without diabetes who took tirzepatide for aesthetic purposes.

## Case presentation

A 23-year-old female was transported to the emergency department with a 4-day history of nausea, vomiting, and diarrhea. She had no history of diabetes, alcohol consumption, or adherence to a carbohydrate-restricted diet. She obtained tirzepatide from an aesthetic clinic without medical supervision and self-administered 2 doses of 2.5 mg weekly for aesthetic weight reduction. Her baseline body weight and height were 58.9 kg and 156 cm (body mass index [BMI] 24.2 kg/m^2^), meeting the World Health Organization (WHO) Asian classification for overweight but not obesity by conventional criteria. After the first injection, she experienced a decrease in appetite and food intake. Three days before presentation, she administered a second dose of tirzepatide, after which she developed severe nausea, vomiting, and diarrhea. As her symptoms did not improve, she was administered intravenous fluids containing glucose at a nearby clinic and was subsequently transferred to our hospital. On arrival, her body weight and BMI had decreased to 53.8 kg and 22.1 kg/m^2^, respectively. Physical examination revealed a pale and fatigued appearance, consistent with prolonged nausea, vomiting, and diarrhea, and a fever of 38.4°C.

## Diagnostic assessment

Laboratory data at the initial presentation are shown in Table 1. Arterial pH was 7.41 [normal reference range (ref): 7.35-7.45] with a serum bicarbonate level of 17.7 mmol/L (ref: 22.0-26.0 mmol/L) and an anion gap of 25.9 mmol/L (ref: 8-16 mmol/L), indicating high anion gap metabolic acidosis with a concomitant metabolic alkalosis likely influenced by vomiting. Serum β-hydroxybutyrate and acetoacetate concentrations were elevated at 5.5 and 1.4 mmol/L (ref: <0.07 mmol/L; <0.01-0.07 mmol/L), respectively. Plasma nonesterified fatty acid (NEFA) levels were increased (2.6 mmol/L) (ref: 0.2-0.55 mmol/L). Plasma glucose and glycated hemoglobin A1c (HbA1c) levels were 196 mg/dL (SI: 10.9 mmol/L) (ref: 70-100 mg/dL [SI: 3.9-5.6 mmol/L]) and 5.5% (ref: 4.6-6.2%), respectively. Serum sodium, potassium, creatinine, and transaminase levels were within normal limits. Rapid antigen tests for influenza and coronavirus yielded negative results. In addition, imaging studies and bacteriological examinations of blood, urine, and stool did not identify a definitive infectious source. Based on these findings, the patient was diagnosed with euglycemic ketoacidosis temporally associated with tirzepatide use.

**Table 1:** Laboratory findings on admission

Variable	Value (Conventional)	Value (SI)	Reference range (Conventional)	Reference range (SI)
pH	7.41	7.41	7.35-7.45	7.35-7.45
HCO_3_^−^	17.7 mEq/L	17.7 mmol/L	22.0-26.0 mEq/L	22.0-26.0 mmol/L
Base excess	−5 mEq/L	−5 mmol/L	−2 to +2 mEq/L	−2 to +2 mmol/L
Anion gap	25.9 mEq/L	25.9 mmol/L	8-16 mEq/L	8-16 mmol/L
β-hydroxybutyrate	57.2 mg/dL	5.5 mmol/L	<0.73 mg/dL	<0.07 mmol/L
Acetoacetate	14.3 mg/dL	1.4 mmol/L	<0.10-0.71 mg/dL	<0.01-0.07 mmol/L
Glucose	196 mg/dL	10.9 mmol/L	70-100 mg/dL	3.9-5.6 mmol/L
HbA1c	5.5%	37 mmol/mol	4.6-6.2%	27-44 mmol/mol
Lactate	27 mg/dL	3.0 mmol/L	4.5-18 mg/dL	0.5-2.0 mmol/L
NEFA	2.6 mmol/L	2.6 mmol/L	0.2-0.55 mmol/L	0.2-0.55 mmol/L
BUN	17.5 mg/dL	6.2 mmol/L	8-20 mg/dL	3.0-7.3 mmol/L
Creatinine	0.89 mg/dL	78.7 µmol/L	0.4-1.1 mg/dL	35-97 µmol/L
eGFR	66.2 mL/min/1.73 m^2^	66.2 mL/min/1.73 m^2^	≥60 mL/min/1.73 m^2^	≥60 mL/min/1.73 m^2^
Sodium	144 mmol/L	144 mmol/L	135-147 mmol/L	135-147 mmol/L
Potassium	4.1 mmol/L	4.1 mmol/L	3.6-5.0 mmol/L	3.6-5.0 mmol/L
Chloride	104 mmol/L	104 mmol/L	98-108 mmol/L	98-108 mmol/L
Total bilirubin	1.1 mg/dL	18.8 µmol/L	0.2-1.2 mg/dL	3.4-20.5 µmol/L
AST	14 U/L	14 U/L	10-33 U/L	10-33 U/L
ALT	12 U/L	12 U/L	6-35 U/L	6-35 U/L
ALP	71 U/L	71 U/L	38-113 U/L	38-113 U/L
γ-GTP	10 U/L	10 U/L	8-60 U/L	8-60 U/L
WBC	18 × 10^3^/µL	18 × 10^9^/L	3.5-9.8 × 10^3^/µL	3.5-9.8 × 10^9^/L
CRP	0.45 mg/dL	4.5 mg/L	<0.35 mg/dL	<3.5 mg/L
TSH	0.2 mIU/L	0.2 mIU/L	0.61-4.23 mIU/L	0.61-4.23 mIU/L
Free T4	1.21 ng/dL	15.6 pmol/L	0.9-1.7 ng/dL	11.6-21.9 pmol/L

Abbreviations: ALP, alkaline phosphatase; ALT, alanine aminotransaminase; AST, aspartate aminotransaminase; BUN, blood urea nitrogen; CRP, C-reactive protein; eGFR, estimated glomerular filtration rate; Free T4, free thyroxine; HbA1c, glycated hemoglobin A1c; NEFA, nonesterified fatty acid; TSH, thyroid-stimulating hormone; WBC, white blood cell; γ-GTP, gamma glutamyl transpeptidase.

## Treatment

The patient initially received intravenous isotonic saline, followed by glucose-containing intravenous fluids with electrolyte supplementation. A continuous intravenous infusion of regular human insulin was administered for 24 hours. On the day after admission, metabolic acidosis improved, and serum ketone body levels rapidly decreased to within the normal reference range; however, nausea and vomiting persisted, likely owing to the sustained pharmacological effects of tirzepatide. The patient also exhibited low-grade fever during the early hospital course, which persisted for several days. In the absence of a definitive infectious source, this fever was considered to reflect physiological stress associated with severe gastrointestinal intolerance and the acute catabolic state, although the contribution of an acute intercurrent illness could not be completely excluded. Intravenous fluid administration was continued for 5 days.

## Outcome and follow-up

The patient's abdominal symptoms gradually improved, and she began regular feeding 4 days after admission; she was discharged 6 days after admission without any medications. At an outpatient follow-up visit 2 weeks later, the patient reported no recurrence of gastrointestinal symptoms and a return to normal appetite. A 75-g oral glucose tolerance test revealed normal glucose tolerance, with a fasting plasma glucose level of 88 mg/dL (SI: 4.9 mmol/L) and a 120-minute value of 101 mg/dL (SI: 5.6 mmol/L) (Table 2). She was advised not to use tirzepatide for aesthetic purposes and agreed to follow this recommendation.

**Table 2:** 75-g oral glucose tolerance test

Time	Plasma glucose	Insulin
0 minutes	88 mg/dL (SI: 4.89 mmol/L)	3.9 µU/mL (SI: 23.4 pmol/L)
30 minutes	136 mg/dL (SI: 7.56 mmol/L)	48.5 µU/mL (SI: 291 pmol/L)
60 minutes	78 mg/dL (SI: 4.33 mmol/L)	23.1 µU/mL (SI: 138.6 pmol/L)
120 minutes	101 mg/dL (SI: 5.61 mmol/L)	25.7 µU/mL (SI: 154.2 pmol/L)

## Discussion

This report describes a rare case of ketoacidosis, clinically manifesting as euglycemic ketoacidosis, in a young woman without diabetes or obesity who self-administered tirzepatide for aesthetic weight reduction. The patient presented with marked appetite suppression, reduced food intake, and severe gastrointestinal symptoms, followed by the development of high-anion gap metabolic acidosis with elevated ketone body levels. The mild hyperglycemia observed was likely attributable to transient stress-related hyperglycemia associated with severe gastrointestinal intolerance and recent administration of glucose-containing intravenous fluids before hospital transfer, rather than underlying glucose intolerance. This glucose level was consistent with the accepted definition of euglycemic ketoacidosis (plasma glucose ≤250 mg/dL [SI: ≤13.9 mmol/L]). Cumulatively, this case adds to the limited but growing body of evidence that euglycemic ketoacidosis may occur in association with tirzepatide use even in nonobese individuals without diabetes, particularly when profound gastrointestinal intolerance and reduced energy intake are present.

Tirzepatide enhances glucose-dependent insulin secretion from pancreatic β-cells, suppresses inappropriate glucagon release, delays gastric emptying, and promotes satiety, reducing blood glucose and body weight [7, 8]. The major adverse effects of tirzepatide are gastrointestinal symptoms, most commonly nausea, vomiting, diarrhea, and constipation [3-7]. These symptoms often lead to decreased appetite, reduced dietary intake, and in some cases, dehydration.

Ketoacidosis associated with tirzepatide has been reported rarely and almost exclusively among individuals with type 2 diabetes or obesity [9-11]. Regarding the dose and timing of tirzepatide, previously reported cases of tirzepatide-associated ketoacidosis occurred at a dose of 5 mg, typically after 3 to 7 injections, with one exception involving concomitant SGLT2 inhibitor use. In contrast, the present patient developed ketoacidosis after only 2 injections at the lowest approved starting dose (2.5 mg), indicating a lower cumulative dose and shorter exposure than in prior reports. Notably, however, the interval between the onset of severe gastrointestinal symptoms and the development of ketoacidosis was several days, comparable to that reported previously. These observations indicate that the development of ketoacidosis may not be determined solely by tirzepatide dose or duration of exposure, but rather by the rapid induction of a profound catabolic state driven by marked appetite suppression and gastrointestinal intolerance.

The pathophysiological mechanism underlying ketoacidosis in the present case appears to be largely consistent with those reported previously and shares key features with starvation-related ketoacidosis. Severe caloric restriction and prolonged gastrointestinal symptoms resulted in an acute catabolic state, which likely played a central role in the development of ketoacidosis. Under such conditions, counter-regulatory hormones such as glucagon and catecholamines increase, enhancing lipolysis and elevating circulating NEFA, which are subsequently converted to ketone bodies in the liver [1, 12].

An important diagnostic consideration in this case is the distinction between starvation ketoacidosis and euglycemic ketoacidosis, which are best understood as existing along a clinical and metabolic continuum. In our patient, several factors likely created a strongly ketogenic milieu. The rapid weight loss of approximately 5 kg over nearly 10 days in a non-obese individual is unlikely to reflect loss of adipose tissue alone and instead indicates an acute and profound negative energy balance accompanied by dehydration and glycogen depletion. Physiological studies have demonstrated that lean individuals may exhibit a more pronounced ketogenic response during fasting than individuals with obesity [12-14], which may have modestly accelerated the development of ketosis under conditions of severe caloric deprivation in this case. However, this interpretation remains speculative, and further studies in larger cohorts are needed to explore whether body composition influences the risk of euglycemic ketoacidosis in this context.

In addition to these starvation-related factors, the presence of mild stress-related hyperglycemia, transient fever, and minimal inflammatory findings at presentation suggests that physiological stress responses further contributed to the ketogenic process. In this context, an acute intercurrent illness, such as viral gastroenteritis, cannot be completely excluded and may have contributed to the physiological stress response and catabolic state, thereby acting as an additional precipitating factor in the development of ketoacidosis. Such stress-related increases in counterregulatory hormones, including catecholamines and glucagon, may have enhanced lipolysis and hepatic ketogenesis. Compared with previously reported cases of ketoacidosis in which hypoglycemia was observed at presentation, these stress-related metabolic responses may have acted as additional exacerbating factors in the present case.

**Figure 1:**
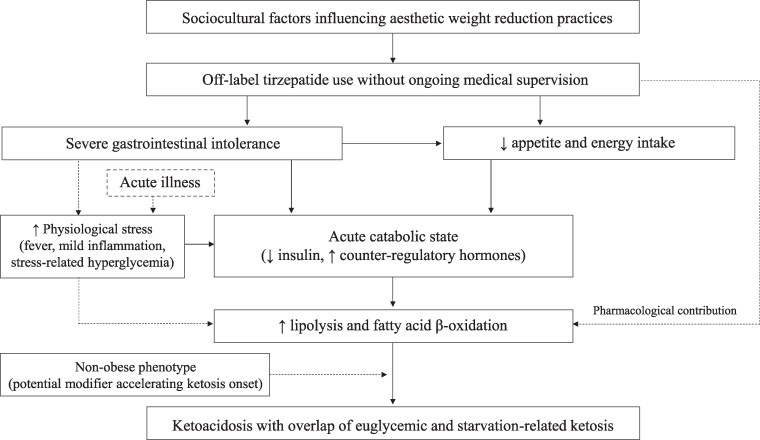
Possible mechanisms contributing to the development of ketoacidosis in this case. Off-label tirzepatide use led to severe gastrointestinal intolerance, reduced appetite and energy intake, and pharmacological metabolic effects. In parallel, an acute intercurrent illness may have contributed to increased physiological stress responses. These factors converged on the development of an acute catabolic state, promoting enhanced lipolysis and fatty acid β-oxidation, and ultimately leading to ketoacidosis with overlapping features of starvation-related and euglycemic ketoacidosis. The nonobese phenotype may have acted as a modifying factor, potentially accelerating the onset of ketosis.

Moreover, pharmacological mechanisms related to tirzepatide may have further modulated the metabolic state. Tirzepatide has been shown to be associated with increased lipid utilization and fatty acid oxidation through its combined GLP-1 and GIP receptor agonism [15]. In particular, GIP receptor activation has been shown to stimulate hormone-sensitive lipase activity in human white adipocytes under fasting conditions, leading to increased NEFA release [16]. In the present case, these pharmacological effects may have acted as contributing or amplifying factors, further enhancing lipolysis and ketone body production. Cumulatively, these findings suggest that the ketoacidosis in this patient arose from the convergence of starvation-related ketosis, stress-induced metabolic responses, and pharmacological modulation of lipid metabolism, rather than a single mechanism alone. These proposed mechanisms are summarized in Fig. 1.

Finally, the attributes of being a young Japanese female and the corresponding sociocultural environment may have influenced the inappropriate off-label use of tirzepatide. Body image distortion and excessive weight control behaviors are common among young females in Japan and other East Asian countries. The International Health and Behavior Survey reported that East Asian females, including Japanese, Korean, and Thai females, tend to perceive themselves as overweight, even within the lower BMI deciles [17]. Studies among Japanese university students and working females have similarly shown that many females with normal BMI consider themselves overweight and attempt unnecessary weight reduction [18, 19]. This sociocultural background may explain the increasing off-label use of tirzepatide for aesthetic weight reduction in Japan, as observed in the present case. Given its potent pharmacological effects and prolonged half-life, this unapproved use poses significant health risks, including malnutrition and ketoacidosis.

This case highlights that ketoacidosis may occur in association with tirzepatide use even in individuals without diabetes or obesity, particularly when severe gastrointestinal intolerance results in acute caloric deprivation. These observations should be interpreted as hypothesis-generating rather than indicative of a generalized drug-related risk. Nevertheless, caution is warranted regarding the off-label use of tirzepatide for aesthetic weight reduction. Increased clinical and public awareness is needed regarding the potential metabolic complications of inappropriate tirzepatide use, especially in non-obese populations where distorted body image and the pursuit of leanness are prevalent.

## Learning points

- Ketoacidosis may occur in association with tirzepatide use, even in individuals without diabetes or obesity, particularly under conditions of marked appetite suppression and severe gastrointestinal intolerance leading to acute caloric deprivation.
- Tirzepatide may enhance fasting-related adipose tissue lipolysis via GIP receptor agonism and promote fatty acid β-oxidation through combined GLP-1 and GIP receptor agonism; these effects are likely contributory rather than causal and may amplify ketosis under conditions of acute caloric deprivation.
- The off-label use of tirzepatide for aesthetic weight reduction warrants caution. Clinicians should promote appropriate use and raise awareness of potential metabolic complications, particularly in nonobese populations where distorted body image and the pursuit of leanness are prevalent.

## Contributors

All authors made individual contributions to the authorship of this manuscript. R.K. and T.O. were responsible for the diagnosis and clinical management of the patient. N.K. contributed to the clinical interpretation of the case. R.I., Y.T., and T.Y. supervised the patient's care and clinical decision-making. All authors reviewed and approved the final draft.

## Data Availability

Data sharing is not applicable to this article, as no datasets were generated or analyzed in the current study.
